# Complete Genome Sequence of Mayaro Virus (*Togaviridae, Alphavirus*) Strain BeAr 20290 from Brazil

**DOI:** 10.1128/genomeA.01372-15

**Published:** 2015-12-17

**Authors:** Danillo Lucas Alves Espósito, Benedito Antônio Lopes da Fonseca

**Affiliations:** Department of Internal Medicine, School of Medicine of Ribeirão Preto, São Paulo, Brazil

## Abstract

We report here the complete genome sequence of Mayaro virus strain BeAr 20290 isolated from *Haemagogus* mosquitoes in 1960. The sequence presented here includes all nonstructural and structural proteins and the 5′- and 3′-untranslated (UTR) regions.

## GENOME ANNOUNCEMENT

Mayaro virus (MAYV) (*Togaviridae*, genus *Alphavirus*) was first isolated in Trinidad in 1954 from blood samples from five febrile patients (1). It is transmitted by an arthropod vector, usually *Haemagogus* sp. mosquitoes, and it is found mainly in South American countries (2). The symptoms of MAYV infection include fever, headache, myalgia, diarrhea, skin rash, and arthralgia (3). Although Mayaro fever is a zoonotic disease, and human cases of infection are sporadic and linked to contact with rainforests, recent demographic changes have been related to an increase in reported cases of MAYV infection in humans (4). Thus, the increasing circulation of MAYV underscores the importance of increasing the knowledge of all characteristics of this virus, including its molecular contents. The MAYV genome consists of a single-stranded positive-sense RNA and encodes four nonstructural proteins (nsP1 to -4) and five structural proteins (C-E3-E2-6k-E1) (5). The genome organization is 5′-nsP1-nsP2-nsP3-nsP4-(junction region)-C-E3-E2-6k-E1-3′.

So far, few complete sequences of MAYV have been submitted to NCBI, and this makes it difficult to perform phylogenetic and molecular analyses of this virus. In Brazil, the strain BeAr 20290, isolated in 1960 from *Haemagogus* mosquitoes, has widely been used as a prototype for this alphavirus infection. Besides that, the genome sequence deposited here represents the first full-length nucleotide sequence of the oldest strain of MAYV isolated in Brazil. Virus samples were passed in Vero cells to increase virus titer, RNA was extracted using silica membrane columns, and cDNA was synthesized using a high-fidelity enzyme. Sequencing was performed in an Ion Personal Genome Machine sequencer (Thermo Fisher) using a second-generation 316 Chip. The Phred quality score of the sequencing was near 85% of bases that were ≥Q20. Contigs were assembled using a previous MAYV genome as a reference in the Geneious R8 software. The full genome presented here has 11,226 bp and an overall G+C content of 51%.

This sequence will certainly be helpful for further investigations of MAYV infections, including diagnostic and phylogenetic analyses of this virus and other alphaviruses.

### Nucleotide sequence accession number.

The complete genome sequence of Mayaro virus strain BeAr 20290 has been deposited in GenBank under the accession no. KT754168.
